# A dynamic application of PRECIS-2 to evaluate implementation in a pragmatic, cluster randomized clinical trial in two nursing home systems

**DOI:** 10.1186/s13063-018-2817-y

**Published:** 2018-08-22

**Authors:** Jennifer A. Palmer, Vincent Mor, Angelo E. Volandes, Ellen McCreedy, Lacey Loomer, Phoebe Carter, Faye Dvorchak, Susan L. Mitchell

**Affiliations:** 1 Harvard Medical School, 25 Shattuck Street, Boston, MA 02215 USA; 2 Hebrew SeniorLife, Institute for Aging Research, 1200 Centre Street, Roslindale, MA 02131 USA; 3 Beth Israel Deaconess Medical Center, Department of Medicine, East Campus, Yamins 419, 330 Brookline Avenue, Boston, MA 02215 USA; 4 Massachusetts General Hospital, Section of General Medicine, 55 Fruit Street Gray 7–730, Boston, MA 02114 USA; 5 Center for Gerontology and Healthcare Research, School of Public Health, Brown University, 121 S Main Street, Providence, RI 02903 USA; 6 Department of Health Services, Policy, and Practice, School of Public Health, Brown University, 121 S Main Street, Providence, RI 02903 USA; 7 Providence Veterans Administration Medical Center, Center of Innovation in Health Services Research and Development Service, 830 Chalkstone Avenue, Providence, RI 02908 USA

**Keywords:** Pragmatic trial, Implementation, PRECIS-2, Nursing homes

## Abstract

**Background:**

PRECIS-2 (PRagmatic Explanatory Continuum Indicator Summary-2) can assess how clinical trial design decisions (along the explanatory-pragmatic continuum) influence the applicability of trial results to intended stakeholders. The tool has been used to assess features of trials during the trial design phase and also upon completion. The ongoing PRagmatic trial Of Video Education in Nursing homes (PROVEN), which is evaluating the effectiveness of a suite of videos to improve advance care planning, is one of the first large pragmatic, cluster randomized trials within nursing home health care systems. While certain features of pragmatic trials remain static once designed (e.g., recruitment, outcomes), successful implementation of a system-wide program requires on-going evaluation and adaptation. This report’s objectives were to apply PRECIS-2 in a novel manner during the actual conduct of the PROVEN trial to assess how dynamic adaptations shifted implementation to either a more explanatory or a more pragmatic approach.

**Methods:**

We assessed PROVEN’s protocol as initially designed according to the three PRECIS-2 domains pertinent to implementation: (1) Organization, (2) Flexibility-Delivery, and (3) Flexibility-Adherence. We then applied this framework to conduct a formative evaluation of decisions made while the trial was ongoing to adapt the implementation approach along the pragmatic versus the explanatory continuum in response to emergent challenges.

**Results:**

Based on the PRECIS-2 rubric, the initial design of the PROVEN implementation approach reflected a hybrid of pragmatic and explanatory features. Most notably, within the Flexibility-Delivery, the trial had a relatively pragmatic approach to protocol delivery by front-line nursing home providers, balanced with a more explanatory approach to protocol monitoring enabled by the analytic capabilities of the research team. This more intensive monitoring proved critical in revealing implementation problems once the study began. Dynamic adaptations made in response to these challenges generally reflected shifts to more explanatory approaches within the Flexibility-Delivery and Flexibility-Adherence domains including ever more intensive compliance monitoring, as well as detailed coaching of front-line providers delivering the intervention by the research team.

**Conclusions:**

Pragmatic trials conducted in the nursing home setting may benefit from a more dynamic approach to implementation. Allowing fluidity between pragmatic and explanatory features may still preserve the trial's applicability to intended stakeholders’ needs. PRECIS-2 provides a useful formative evaluation tool to assess these adaptations in “real-time.”

**Trial registration:**

US National Library of Medicine, ClinicalTrials.gov, ID: NCT02612688. Registered on 19 November 2015

## Background

Pragmatic, cluster randomized clinical trials (RCTs) are increasingly employed in health services research to test the effectiveness of interventions under “real-world” conditions [1]. The inherent tension in designing and conducting pragmatic trials is the need to adhere to a rigorous clinical trial design while maintaining study conditions as closely as possible to usual practice. Central to this challenge is the study’s implementation strategy.

In the past 30 years, nursing homes have evolved into complex health care systems serving increasingly sick older patients [2, 3]. Many nursing homes in the USA exist within large corporate chains, sharing common organizational, health information, and clinical infrastructures. Thus, they are well-suited for implementing interventions within a pragmatic clinical trial. However, very little is known about how best to implement aspects of pragmatic trials in nursing home facilities, as very few trials of this kind have been conducted.

The PRagmatic trial Of Video Education in Nursing homes (PROVEN) is an ongoing study funded by the National Institutes of Health and one of the first large pragmatic trials in the nursing home setting [4]. It is being conducted in partnership with two large health care corporations in 360 nursing facilities across the USA. The over-riding objective of PROVEN is to evaluate the real-world effectiveness of an Advance Care Planning (ACP) Video Program to improve goal-directed decision-making by frail nursing home residents or their family members. In the spirit of a pragmatic trial, the research team (RT), in close collaboration with their health care system partners, strove to design the intervention implementation approach to align as closely as possible with how new programs are typically rolled out and evaluated within these corporations. Implementation began in March 2016 and was scheduled to be completed in May 2018. Since implementation started, many of the pragmatic trial features of the original protocol have worked well, while others have met challenges requiring ongoing adaptation while implementation is in progress.

PRECIS (PRagmatic Explanatory Continuum Indicator Summary) is a tool employed to optimize a trial’s applicability to the needs of intended end-users. Some end-users need results from pragmatic trials (that test interventions as they would be enacted in the “real world”) versus explanatory trials (that test interventions under “idealized” circumstances). PRECIS helps investigators evaluate and align their clinical trial design accordingly [5]. A revision of the original PRECIS, PRECIS-2, includes nine domains: (1) Eligibility, (2) Recruitment, (3) Setting, (4) Organization, (5) Flexibility-Delivery, (6) Flexibility-Adherence, (7) Follow-up, (8) Primary Outcome, and (9) Primary Analysis. The Organization, Flexibility-Delivery, and Flexibility-Adherence domains are the most pertinent for assessing a trial’s approach to implementation. While PRECIS-2 includes a scale that rates each domain from 1 (most explanatory) to 5 (most pragmatic) [6], the experience of prior investigators suggests that the tool may be more useful as a general framework for discussion rather than as a rating system [7].

Thus, this report leverages the relevant PRECIS-2 domains to evaluate PROVEN’s original pragmatic implementation strategy along the pragmatic versus the explanatory continuum and to discuss decisions that were made to adapt this strategy along this continuum. While prior publications have used PRECIS-2 to evaluate pragmatic RCTs [7–10], our report is novel in several ways. First, it is the first to apply PRECIS-2 to a pragmatic RCT conducted in a nursing home health care system. Second, it focuses exclusively on PRECIS-2 implementation domains. Finally, we conduct this formative evaluation mid-implementation rather than in either the trial’s planning phase or after completion. This last aspect represents the most salient contribution of this work, as it is well-recognized that successful implementation of a system-wide program in the nursing home setting requires on-going evaluation and adaptation [11].

## Methods

The key elements of the initial PROVEN trial design necessary to understand its implementation strategy are presented, followed by a brief description of the three PRECIS-2 domains relevant to implementation.

### Trial overview

The study’s conduct was approved by the Institutional Review Board at Brown University. The details of the overall PROVEN protocol can be found elsewhere [4]. Briefly, PROVEN was initially designed as a pragmatic, cluster RCT seeking to evaluate the real-world effectiveness of an ACP Video Program intervention, targeted to patients or their family members, compared to usual ACP practices (control). As a pragmatic trial, the intent was to shed light on the generalizability of the intervention beyond homogenized, researcher-controlled settings. Thus, trial results could inform health care system (HCS) leaders and policymakers as to the intervention’s value in improving ACP in the nursing home setting.

The trial is being conducted in partnership with two for-profit nursing home HCS that together operate 456 facilities (HCS1, *N* = 358; HCS2, *N* = 98) in 32 states. Eligible facilities met the following criteria: (1) had over 50 beds, (2) served both short- and long-term patients, and (3) lacked serious organizational or regulatory compliance problems based on the perspective of corporate leaders. The eligible 360 facilities were randomly assigned to either an intervention or a control arm (total: *N* = 119 intervention/*N* = 241 control; HCS1: *N* = 98 intervention/199 control; HCS2: *N* = 21 intervention/42 control). Corporate leaders informed administrators at facilities randomized to the intervention arm that their nursing homes were selected to participate in the ACP Video Program, and all agreed to participate.

As the ACP Video Program is implemented facility-wide, all patients in the intervention and control facilities during the planned18-month implementation period comprise the study population. However, for analytic purposes, the primary effectiveness outcome will compare the number of hospital transfers/person-day alive over 12 months among a target population of long-stay residents with advanced dementia or cardiopulmonary disease in the intervention versus control nursing homes. Analysis was planned according to intention-to-treat principles, such that all randomized facilities will be included in the analyses. All population characteristics and outcome data were/will be ascertained from existing administrative and clinical databases including the Minimum Data Set (MDS) [12–14] and Medicare claims data. No restrictions were placed on other ongoing ACP activities (e.g., use of Medical Orders for Life-Sustaining Treatment (MOLST) forms) in either the intervention or control facilities.

### Intervention structure

The ACP Video Program consists of five videos developed by the RT, which have been shown to improve ACP planning in several small traditional RCTs [15–20]. The videos are 6 to 10 min in duration and cover ACP decisions commonly addressed by the nursing home population: **(**1) General Goals of Care, (2) Goals of Care for Advanced Dementia, (3) Hospice, (4) Hospitalization, and (5) Advance Care Planning for Healthy Patients**.** Narrative explanations and visual images are presented. For this trial, the videos were pre-loaded onto tablet devices (two devices per facility) and also accessible to patients and families through a website.

### Intervention implementation and training

The planned 18-month implementation period began on 1 March 2016 in staggered waves (approximately 30 intervention facilities/wave) with all facilities on board by 1 June 2016. The implementation strategy was collaboratively designed by the RT and corporate leaders but executed primarily by the HCS by leveraging existing infrastructures for new program roll-out. Each HCS assigned a corporate-level administrator to broadly oversee the project and, with funding from the research grant, also hired a 50% full-time equivalent clinical education specialist specifically dedicated to leading program implementation. At each facility, one or more providers were designated as ACP champions to lead on-the-ground implementation. One principal investigator (AEV) and one full-time project director on the RT were dedicated to the implementation effort.

The initial implementation strategy employed both standardized and flexible components as made explicit in the training materials and activities. The protocol standardized the timing of video offering: new admissions or re-admissions had to be offered a video within 7 days of arrival at the facility, and long-stay patients (length of stay > 100 days) had to be offered a video every 6 months. The protocol was flexible with respect to the discipline of the provider who should offer the videos but suggested the individuals typically responsible for ACP activities. In almost all circumstances, social workers assumed this role and also served as ACP champions.

The initial protocol provided guidance about which video to show to which patient or proxy, but the ultimate choice was left to the providers based on each patient’s circumstance. For example, the General Goals of Care video was suggested for most patients; however, if the patient had advanced dementia, it was suggested their proxy should be offered the Advanced Dementia Goals of Care video. The provider could also decide who should view the video: patient, proxy, or both. Finally, while providers were encouraged to show the videos on the tablets at the nursing home, they could give the patients/family members a website URL to access the videos at a later time.

Training materials were created and supplied by the RT. HCS leaders provided input into the contents and co-led the training sessions with the RT. Training occurred during the month prior to implementation for each wave of nursing homes. Training provided guidance on the standardized and flexible aspects of the implementation protocol. Training also included suggestions on how to integrate the videos into existing ACP counseling practices. ACP champions were encouraged to customize the ACP Video Program to their local culture and work flow.

Upon PROVEN’s roll-out, monthly telephone group “check-in” conference calls were held among the approximately 30 ACP champions from each nursing home implementation wave, the HCS’ clinical education specialist, and the research implementation team. These calls provided opportunities to review the progress of implementation, share experiences among ACP champions, present challenges, and problem-solve solutions.

### Protocol delivery and adherence monitoring

To monitor protocol delivery and adherence, a patient-level Video Status Report (VSR) was designed and integrated into the HCS’ electronic medical record systems. ACP champions were instructed to complete a VSR each time a video was offered, even if it was not shown. If the video was not shown, the reason was recorded (e.g., patient/family refused). If a video was shown, the report also documented which video(s) was/were viewed and who watched it (e.g., family, patient). For video views on the website (versus the tablets), the RT could track views aggregated at the HCS level but not at the facility or person level.

During the initial study design phase, investigators and corporate leaders carefully weighed whether the appropriate measure of protocol compliance should be “offering” or “showing” a video. Offering was chosen as it was felt to be more “pragmatic” in that, in usual practice, a patient or family member could refuse to engage in ACP activities offered to them.

Two types of feedback reports were developed to assess protocol compliance during implementation. First, the RT planned to link VSRs and HCS-based MDS data to generate quarterly reports that showed, both for each facility and for all intervention facilities in the HCS: (1) the proportion of patients with VSR completed (i.e., indicating that a video was offered) within 7 days of admission/re-admission and (2) the proportion of long-stay patients who ever had a completed VSR.

Second, the HCS planned to generate simpler, internal feedback reports every 2 weeks, which only included the proportion of new or re-admissions who had a completed VSR. These internal reports would be shared at the monthly ACP champion group “check-in” calls to identify and strategize with low-performing facilities about ways to improve protocol delivery.

A final source of intended monitoring included qualitative telephone interviews (approximately 15 min each) with the ACP champions that were conducted by the RT at 4, 9, and 15 months into the implementation period. Areas explored during these interviews included: (1) concomitant ACP activities other than the ACP Video Program (e.g., use of MOLST forms), (2) the ACP champion’s experience with the ACP Video Program training, and (3) the ACP champion’s views on the experience of implementing the ACP Video Program (e.g., barriers, facilitators, reactions of families, patients, physicians, and nurses). These interviews were mainly meant to evaluate implementation upon the trial’s completion; however, they would ultimately provide “real-time” insights that led to modifications of the implementation protocol as described below.

### PRECIS-2

Below we describe the general considerations under each of the three PRECIS-2 domains that focus on implementation: (1) Organization, (2) Flexibility-Delivery, and (3) Flexibility-Adherence [6]. In applying the PRECIS-2 terminology to PROVEN, we define “usual care or conditions” as how a new program is typically rolled out in a nursing home HCS outside of the context of a clinical trial.

The Organization domain considers what personnel, training, and other resources were required to deliver the intervention. In a highly pragmatic trial, the research infrastructure would minimize the supply of any personnel, training materials and venues, or other resources (e.g., devices, technology platforms) necessary to implement the intervention. Rather, these organizational elements would be provided by the HCS in which the new program was being implemented as in real-world circumstances.

The Flexibility-Delivery domain considers the following: (1) the degree to which the implementation was driven by a prescribed protocol, (2) the extent to which the compliance of providers delivering the intervention was monitored, and (3) the extent to which co-interventions outside of the trial were permitted. A more pragmatic design would allow greater flexibility in protocol delivery (e.g., timing, who implements it), whereas a more explanatory trial would involve greater control by the RT. A highly pragmatic trial would also not introduce measures to monitor and improve provider compliance with protocol delivery beyond what would happen under usual conditions. Finally, a highly pragmatic trial would also not restrict the concurrent use of other activities potentially related to the trial intervention (e.g., the MOLST program).

The Flexibility-Adherence domain considers trial design features related to whether or not end-users ultimately engage with the interventions as intended. In PROVEN, this domain may be interpreted as approaches taken to ensure that patients and families viewed a video. This domain also considers the extent to which potentially non-adherent nursing homes were excluded from trial eligibility and the handling of non-adherent participating facilities once implementation is underway. A highly pragmatic trial design would not restrict enrollment or ongoing participation of less adherent sites.

The Flexibility-Adherence domain also considers the degree to which trial-specific adherence monitoring measures were put in place. Given that, in PROVEN, monitoring of delivery (did providers comply with the protocol) was so intertwined with monitoring of adherence (did patients and family view a video?), we discuss both aspects of monitoring under the Flexibility-Delivery section for ease of presentation.

## Results

Using the three outlined PRECIS-2 domains, we assess where PROVEN’s original implementation strategy fell along the pragmatic-explanatory continuum and how that approach has evolved during implementation, which is still on-going. (Table 1).

**Table 1:** PRECIS-2 domains of implementation in the PROVEN trial: approaches and challenges

Domain^a^	Aspect^a^	Approach as originally designed	Challenges	Approach/adaptation
Organization	Personnel	RT: one principal investigator and one project director on intervention team; data team created feedback reports HCS: one corporate-level administrator and one clinical education specialist oversaw implementation; ACP champion(s) delivered intervention at NH; information technology personnel integrated VSR into EMR	• Turnover of HCS clinical education specialists • ACP champion turnover	• Redundancy in HCS leadership roles • Creates detailed tracking system for champions • Regular trainings for champion replacements
	Resources	RT: developed videos; supplied tablet devices; created website URLs for viewing videos HCS: provided EMR system to host VSR	• Two NHs had mostly Navajo patients • Tablets missing at one NH	• RT created translated videos • RT replaced missing tablets
	Training	RT: developed training materials HCS: organized and provided training venues RT and HCS: co-led trainings	• Each HCS had different preferred modalities	• HCS1 webinar-based training; HCS2 group on-site training
Flexibility (delivery)	Protocol-driven	RT: prescribed guidelines for when to offer video; flexible guidelines for which video to offer, who shows videos, how to show (tablet vs. website URL) HCS: ACP champions delivered intervention	• Limited control of how champion implemented protocol • Competing champion clinical responsibilities	• HCS leaders strongly endorsed program • Champion support and coaching
	Monitoring (did providers deliver the intervention per protocol?)	HCS: embedded VSR in EMR; internal bi-weekly feedback reports for VSR completion on new or re-admissions only RT: created VSR; generated quarterly feedback reports for new or re-admissions and LTC; ACP champion qualitative interviews RT and HCS: monthly group champion check-in calls	• Poorer delivery to LTC patients vs. new or re-admissions • Delays and inaccuracies in RT generated reports • VSR seen as a barrier • Group check-in calls inefficient	• Champions retrained • RT feedback report generated monthly • 1:1 champion calls replace group calls • Enrollment period extended
	Co-interventions	RT and HCS: other on-going initiatives to improve ACP activities and reduce hospitalizations allowed	• Other ACP activities variable & not easily measured • Hospitalization rates drop	• Questions about ACP activities inserted into champion interviews
Flexibility (adherence)	Pre-screening	RT and HCS: excluded NHs with major organizational or regulatory difficulties	• Determination of “dysfunctional” sites was subjective	–
	Withdrawal	RT and HCS: NHs with low adherence were *not* dropped	• No uptake in ~ 10% of NHs • Small number of NHs divested mid-study	• Intention-to-treat analyses
	Monitoring (did patients/families view videos as intended?)	RT: protocol compliance initially defined as VSR completion (i.e., offering a video) vs. showing a video; analysis of VSR items able to examine whether or not video was shown when offered	• Videos commonly not shown when offered • Web-based video viewing rates could not be tracked	• Showing rates added to feedback reports • LTC patients not shown a video identified and targeted through intense champion coaching

*RT*  research team, *HCS*  health care system, *LTC* long-term care, *NH* nursing home, *ACP* advance care planning, *EMR* electronic medical record, *VSR* video status report

^a^PRECIS-2 Domains and Aspects derived from Loudon et al. (2015) [6]

### Organization

The personnel involved in PROVEN’s implementation reflected a dynamic blend of pragmatic and explanatory features. The “on-the-ground” implementation activities were continuously led by HCS personnel. HCS corporate-level administrators and clinical education specialists directed the facility ACP champions in implementing the program, and it was these champions, not the RT, who offered videos to patients and families. The RT’s initial involvement was largely “behind-the scenes,” such as designing the implementation and training protocols and creating feedback reports. However, as will be discussed below, once challenges with protocol compliance were revealed early in the implementation period, one of the RT’s PI (AEV) took an increasingly active role in coaching ACP champions, albeit always in partnership with the HCS clinical education specialists.

Turnover of ACP champions was the main challenge within the Organization domain. It prompted intensive tracking of the champions at each facility by the HCS clinical education specialist, as well as training of new champions as needed. The clinical education specialist position was also vacated and re-filled one time in each HCS during the implementation period. The potential disruption to implementation by these transitions was somewhat attenuated by the fact that that the corporate-level project leaders were very familiar with the program and could temporarily fill-in and ultimately train the new clinical education specialists.

The trial resources were more aligned with an explanatory trial, as the RT created the videos, purchased, pre-loaded, and distributed the tablet devices, and made the videos accessible via a website URL. In the real world, the HCS would have had to acquire these resources independently in order to implement an ACP Video Program. The HCS supplied the electronic medical record system that hosted the VSR, as well as information technology personnel who enabled that effort. The few problems encountered with project resources were handled by the RT. For example, the RT created translated and customized versions of the General Goals of Care video for two facilities that served mostly Navajo-speaking patients; also, lost tablets were replaced in another facility.

Training activities, also under the Organization domain, reflected a hybrid of explanatory and pragmatic features. The RT supplied all the training materials and primarily designed the training sessions. However, training activities leveraged each HCS’ particular existing infrastructure used to roll out new programs. The larger, national organization, HCS1, opted for training via Intranet-hosted webinars, which is the standard approach that they use for uniform training. On the other hand, the smaller, regional organization, HCS2, opted for centralized, in-person trainings for which they provided the venue. This approach was customary, since they regularly bring staff together for training.

### Flexibility-Delivery

Within the Flexibility-Delivery domain, protocol delivery was mostly pragmatic. While the RT designed the protocol, it had limited control over its delivery, which was largely dependent on ACP champions’ discretion. The main prescribed element in the protocol was that providers should offer a video to all new or re-admissions and every 6 months to long-term care patients.

The trial’s monitoring mechanisms for both protocol delivery (i.e., did the ACP champions offer a video as per protocol?) and adherence (i.e., did the patients or family members actually view a video?) were decidedly less pragmatic but proved essential in revealing major implementation problems in a timely manner. The VSR was created by the RT as a “new” data source specifically for the trial and, thus, reflects a relatively more “explanatory” feature. Each HCS integrated the VSR into its electronic medical record system and used it to generate internal feedback reports, which supports the use of this monitoring approach under real-world conditions. However, the RT’s more robust analytic capabilities were ultimately needed to detect implementation problems. Most notably, by linking the VSR with HCS-based MDS resident assessment data, the RT could monitor VSR completion for both new admission and long-stay cohorts (i.e., delivery monitoring). Moreover, the RT could calculate the rates at which videos were actually shown (i.e., adherence monitoring) not just offered. The ACP champion qualitative interviews were another source of monitoring that would not have occurred outside of a trial and inadvertently revealed challenges to protocol delivery (i.e., ACP champion turnover, lack of attention to the long-stay residents). Taken together, the extent and formality to which protocol delivery and adherence monitoring occurred in PROVEN may not have occurred under real-world conditions.

The aforementioned monitoring mechanisms revealed three major implementation problems: (1) the overall rate of VSR completion (i.e., video offering) was much lower than anticipated, (2) the offering rate was particularly low among long-stay residents compared to new or re-admissions, and (3) in a high proportion of cases, videos offered were not actually shown. During monthly group “check-in” calls, ACP champions cited lack of time and competing responsibilities as the biggest barriers to not offering the video at all. They also described situations in which they felt it inappropriate to offer a video because the patient’s care plan already indicated a preference for comfort care (e.g., enrolled in hospice care), a circumstance that was not captured on the VSR. In fact, the VSR itself proved to be a hindrance to protocol delivery, as ACP champions found it did not serve any clinical purpose and, thus, often did not complete it.

The relatively better protocol delivery among new or re-admissions most likely reflected that it was more straightforward to integrate the video offering into the work flow of the distinct event of an admission rather than the more nebulous instructions to extend a video offer every 6 months to the long-stay group. While regularly scheduled care planning meetings were suggested as potential trigger events to offer videos in the long-stay cohort, often neither family members nor patients attended these meetings; when they did, ACP champions stated there was often not enough time to show a video.

The major problem with adherence (i.e., patients and families not actually viewing the videos) was an unintended consequence of our initial decision to define protocol compliance as “offering” rather than “showing” a video, a definition that was reinforced in the initial training of ACP champions and in the feedback reports provided to them. Thus, this phenomenon may have reflected an attempt by the champions to meet compliance benchmarks in the easiest manner possible.

Attempts to improve protocol delivery primarily involved the clinical education specialists reinforcing with ACP champions that delivery of the ACP Video Program was an expected responsibility. Reinforcement was achieved through joint review of the feedback reports, monthly group check-in calls, and on-site visits to poor performing facilities. These initial efforts met with limited success. Subsequent steps that ultimately proved critical in improving protocol delivery required a shift towards a more explanatory approach. First, the frequency of providing feedback reports to ACP champions was increased from quarterly to monthly, and the report content was modified to include the rate of showing, not just offering, videos. Second, ACP champions were told that, going forward, they would be held accountable for showing (versus offering) videos. They were also instructed to shift their efforts more towards long-stay residents relative to new or re-admissions since the trial’s primary outcome would be measured in this cohort. To increase the focus on showing videos to this target population, the research data team generated for every facility a list that indicated which long-stay residents had not yet seen a video. The PROVEN implementation team leaders then conducted monthly telephone meetings with each facility ACP champion, and together they identified who among these long-stay residents were most in need of advance care planning and strategized how to facilitate those residents or their family members viewing a video.

As an additional step to improve intervention adherence, after consultation with internal and external statistical experts, the subject enrollment period was extended from the planned 18 months to 24 months with the hope that, with the modifications to the implementation protocol, a greater number of long-stay residents would be exposed to the videos. This extension incurred a small increase in research funds to support the on-going efforts of the HCS partners, resources that would not necessarily be available under real-world conditions.

Finally, within the Flexibility-Delivery domain, PROVEN was entirely pragmatic in regards to co-interventions, as it allowed on-going ACP activities (e.g., use of MOLST forms) or programs to reduce hospitalization rates to occur alongside the trial. Although information about these co-interventions was ascertained in the intervention facilities during the ACP champion qualitative interviews, the presence of co-interventions could not be known in the control nursing homes. Thus, the impact of differential use of co-interventions between arms on the trial’s outcomes would not be directly measurable, a limitation of a pragmatic approach but also reflective of what happens in the real world.

### Flexibility-Adherence

In the Flexibility-Adherence domain, in terms of “pre-screening” potentially non-adherent sites, the RT asked the HCS at the outset of the trial to eliminate facilities with major organizational or regulatory difficulties from the pool of eligible nursing homes. In line with intention-to-treat principles, all randomly allocated nursing homes will be retained in the analyses, including the approximately 10% of intervention nursing homes that displayed little to no adherence to the protocol and several nursing homes that were divested from HCS1 after the start of implementation. This approach is pragmatic in nature and will be enabled by the fact that follow-up and outcome information from non-adherent sites are universally available on all residents from existing sources (i.e., the MDS and Medicare claims).

## Discussion

In this report we demonstrate that, with a few caveats, the three implementation-focused PRECIS-2 domains provide a useful framework to evaluate the implementation strategy of a large cluster pragmatic RCT conducted in the nursing home setting. Our formative evaluation approach further extends the utility of the PRECIS-2 tool by applying it while the implementation is actually in progress rather than during the design phase or upon trial completion [7–10]. In general, we find that evolution from an intended pragmatic trial to a hybrid of pragmatic and explanatory features is necessary for successful implementation in this complex heath care setting but in a manner that is dynamically responsive to challenges encountered while in the field.

Prior studies have used the PRECIS-2 tool to evaluate pragmatic RCTs in various settings, [7, 8, 10] albeit not in nursing homes and not while the trial was in progress. Nonetheless, similar to our experience, these earlier studies found that most pragmatic trials have both pragmatic and explanatory features but that the PRECIS-2 implementation domains tend to be more explanatory compared to other domains [7, 10].

A seemingly more explanatory aspect of a trial does not mean that it is less pragmatic, however. This is an important point when considering the applicability of trial results to the real-world needs of health care system stakeholders. That is, a more explanatory approach could be adopted by an organization that opts to roll out a system-wide clinical or quality improvement program in the real world. For example, while the PROVEN RT supplied key resources (e.g., videos, tablet devices), these elements are certainly attainable by a HCS committed to delivering an ACP Video Program outside of a trial. Moreover, almost all the more explanatory aspects of PROVEN’s approach (e.g., monitoring) can conceivably be assumed by a nursing home HCS outside of a rubric of a research trial (e.g., through a mandated standard operating procedure) and, thus, are not necessarily “non-pragmatic.”

Our work has revealed this need for fluidity between pragmatic and explanatory aspects of implementation. In PRECIS-2’s Organization domain, PROVEN’s implementation required increasingly greater involvement of the RT; however, maintenance of a high level of collaboration between the investigators and HCS’ project leaders was critical to implementation. This established approach can be characterized as blended facilitation in which internal (i.e., HCS clinical education specialists, HCS administrative leaders, ACP champions) and external (i.e., researchers) facilitators to an organization are engaged in shared support and problem-solving that optimize each partners’ respective strengths [21]. Also in the Organization domain, the high turnover of ACP champions is clearly a limitation but also a well-recognized and unavoidable real-world problem within the nursing home industry, which we attempted to minimize through close tracking of staff, redundancy in facilitator roles, and frequent training sessions.

PROVEN has also demonstrated that a highly pragmatic approach to protocol delivery must be balanced with a more explanatory approach to protocol monitoring. Within the Flexibility-Delivery domain, even a relatively straightforward intervention, such as the ACP Video Program, faces implementation challenges similar to those in more complex nursing home programs [22, 23], such as competing demands of front-line providers. Only by the RT’s production of quantitative feedback reports and conduct of qualitative interviews with ACP champions were critical gaps in implementation revealed. The PROVEN experience to date also reflects the potential need to modify the pragmatic-explanatory balance of the monitoring approach after implementation has begun. For example, once we detected low adherence rates, we adapted the protocol such that our primary measure of compliance shifted from “offering” to “showing” a video.

This work is descriptive in nature. We did not apply PRECIS-2 to inform adjustments of our implementation strategy but rather examined these adjustments shortly thereafter (still in “real-time”) to frame lessons learned and advance the science regarding pragmatic trials. Regardless, our efforts highlight the value of PRECIS-2 as a formative evaluation tool which could be more directly used to influence implementation strategies mid-trial in future research.

## Conclusions

Taken together, this report suggests that pragmatic RCTs conducted in the nursing home setting may benefit from a more dynamic approach to implementation which allows for fluidity between pragmatic and explanatory features. Such fluid designs can still maintain the applicability of trial results to real-world stakeholders. Pragmatic and explanatory designs are not dichotomous, and we would argue that nursing home systems can often implement relatively “explanatory” efforts while sustaining real-world viability. In contrast, other PRECIS-2 domains (i.e., Eligibility, Recruitment, Setting, Primary Outcome, and Primary Analysis) must be relatively more adherent to their initial design. As the PROVEN trial continues towards completion, it remains to be assessed whether adaptations in the implementation approach improve the uptake of the ACP Video Program such that its effectiveness can be demonstrated and whether the program remains feasible outside of a clinical trial.

## Abbreviations

ACP: Advance care planning
EMR: Electronic medical record
HCS: Health care system
LTC: Long-term care
MDS: Minimum Data Set
MOLST: Medical Orders for Life-Sustaining Treatment
NH: Nursing home
PRECIS: PRagmatic Explanatory Continuum Indicator Summary
PROVEN: PRagmatic trial Of Video Education in Nursing homes
RCT: Randomized clinical trial
RT: Research team
VSR: Video Status Report
